# The structure of *Medicago truncatula* δ^1^-pyrroline-5-carboxylate reductase provides new insights into regulation of proline biosynthesis in plants

**DOI:** 10.3389/fpls.2015.00869

**Published:** 2015-10-30

**Authors:** Milosz Ruszkowski, Boguslaw Nocek, Giuseppe Forlani, Zbigniew Dauter

**Affiliations:** ^1^Synchrotron Radiation Research Section, Macromolecular Crystallography Laboratory, National Cancer InstituteArgonne, IL, USA; ^2^Biosciences Division, Argonne National Laboratory, The Structural Biology CenterArgonne, IL, USA; ^3^Department of Life Science and Biotechnology, University of FerraraFerrara, Italy

**Keywords:** protein structure, decamer, coenzyme preference, salt stress, abiotic stress, P5C reductase, P5CR

## Abstract

The two pathways for proline biosynthesis in higher plants share the last step, the conversion of δ^1^-pyrroline-5-carboxylate (P5C) to L-proline, which is catalyzed by P5C reductase (P5CR, EC 1.5.1.2) with the use of NAD(P)H as a coenzyme. There is increasing amount of evidence to suggest a complex regulation of P5CR activity at the post-translational level, yet the molecular basis of these mechanisms is unknown. Here we report the three-dimensional structure of the P5CR enzyme from the model legume *Medicago truncatula* (*Mt*). The crystal structures of unliganded *Mt*P5CR decamer, and its complexes with the products NAD^+^, NADP^+^, and L-proline were refined using x-ray diffraction data (at 1.7, 1.85, 1.95, and 2.1 Å resolution, respectively). Based on the presented structural data, the coenzyme preference for NADPH over NADH was explained, and NADPH is suggested to be the only coenzyme used by *Mt*P5CR *in vivo*. Furthermore, the insensitivity of *Mt*P5CR to feed-back inhibition by proline, revealed by enzymatic analysis, was correlated with structural features. Additionally, a mechanism for the modulation of enzyme activity by chloride anions is discussed, as well as the rationale for the possible development of effective enzyme inhibitors.

## Introduction

In plant cells the function of proline goes much further than being a building block for proteins (Szabados and Savouré, 2010). Many studies have shown that free proline can trigger signal transduction pathways associated with different stress responses. The signaling function of proline was first observed in bacteria and was related to osmotic stress (Csonka, 1988; Csonka et al., 1988; Csonka and Hanson, 1991). Later on, proline was also reported to be a key player in plant adaptation to adverse environmental conditions (Verslues and Sharma, 2010), such as high salinity (Yoshiba et al., 1995), drought (Choudhary et al., 2005), abnormal doses of UV radiation (Saradhi et al., 1995), exposure to heavy metals (Schat et al., 1997), reactive oxygen species (Yang et al., 2009), and pathogens (Fabro et al., 2004; Haudecoeur et al., 2009). Interestingly, it was recently reported that free proline can also regulate development, flowering and reproduction (Mattioli et al., 2008, 2009; Funck et al., 2012).

**Figure 1:**
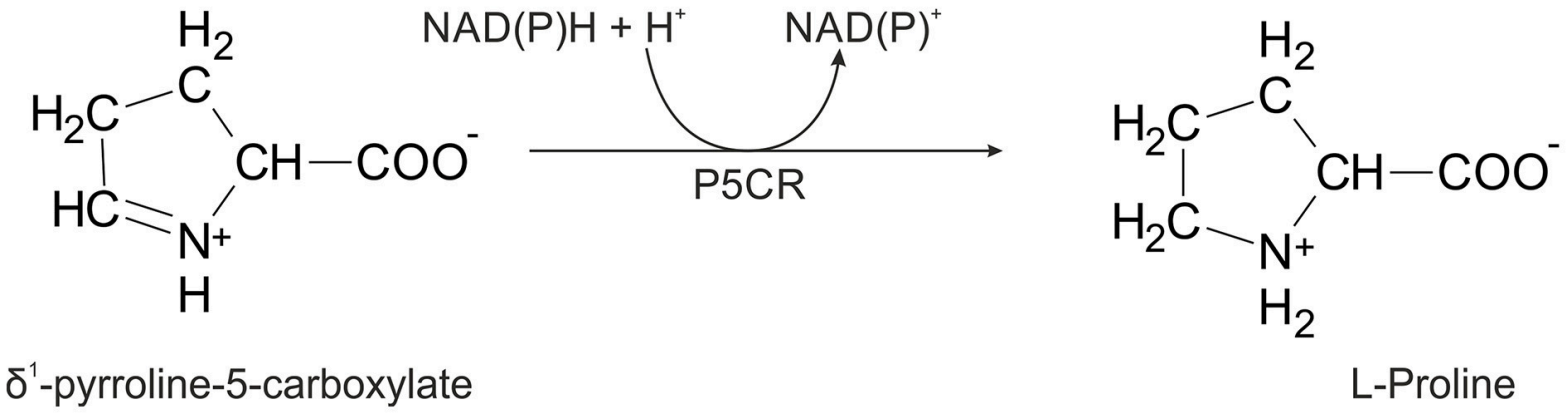
**Scheme of the enzymatic reaction catalyzed by P5CR**.

In higher plants, proline can be synthesized *via* two main pathways, one starting from glutamate (Smith et al., 1980; Székely et al., 2008) and the other from ornithine (Abdelal, 1979; da Rocha et al., 2012). The first route, localized either in the cytoplasm or in the chloroplast (Lehmann et al., 2010), involves phosphorylation of glutamate to γ-glutamyl phosphate and its reduction to glutamate γ-semialdehyde (GSA), which undergoes spontaneous, non-enzymatic cyclization to δ^1^-pyrroline-5-carboxylate (P5C). The two enzymatic steps are catalyzed by a bifunctional P5C synthetase (P5CS; EC 2.7.2.11/1.2.1.41). In the second pathway, ornithine is directly transformed into GSA/P5C by a mitochondrial ornithine δ-aminotransferase (OAT; EC 2.6.1.13; Winter et al., 2015). The pathway preference appears to depend on nitrogen availability, being the ornithine route preferred under nitrogen-rich conditions (Delauney and Verma, 1993; da Rocha et al., 2012). Both routes converge at the generation of P5C, which in the final step (Figure 1) is reduced to L-proline by P5C reductase (P5CR, EC 1.5.1.2). This means that P5CR is always required for proline biosynthesis. Consistently, null mutations in *p5cr* genes are embryo lethal (Funck et al., 2012), and targeting specific inhibitors of P5CRs is of great interest as a solution for weed control (Forlani et al., 2007, 2013).

Salt and drought stress-induced proline synthesis plays a main role in legume plants, where both the ornithine (through enhanced transcription of *OAT*) and the glutamate pathways (*P5CS isozyme 2*) contribute to proline accumulation (Armengaud et al., 2004). Under osmotic stress conditions, the efficiency of establishment of the *Rhizobium*–legume symbiosis and the rate of nitrogen fixation are dramatically affected, reducing crop yield, and soil-enriching capacity (Coba de la Peña et al., 2003). Interestingly, high proline concentrations have been found in fava bean symbiosomes, which significantly increased in response to treatments with NaCl (Trinchant et al., 1998). Stress-induced proline accumulation was higher in the peribacteroid space, whereas very low rates of proline uptake by bacteroids were found, suggesting a role for proline as osmoticum rather than energy source for bacterial symbionts (Trinchant et al., 1998). Consistently, proline overaccumulating transgenic *Medicago truncatula* plants displayed nitrogen-fixing activity with enhanced tolerance to osmotic stress (Verdoy et al., 2006). Moreover, higher P5CR activity in ureide-exporting than in amide-exporting nodules led to hypothesize that NADP^+^ production, deriving from the reduction of P5C, may be also functional to fuel the oxidative pentose phosphate pathway (OPPP), which is required for the synthesis of ureides (Kohl et al., 1990). The complexity of proline metabolism and its regulation in legume plants is witnessed by the discovery in *M. truncatula* of a third gene coding for a P5CS isozyme, which has been shown to play a predominant role in stress-induced proline accumulation during symbiotic nitrogen fixation (Kim and Nam, 2013).

While the three-dimensional structures of human and some bacterial P5CR orthologs have been determined, to date high-resolution crystal structures of plant P5CRs have been absent. This hampered the possibility of deeper understanding of enzyme features and post-translational regulative mechanisms. Indeed, plant P5CRs have been recently found to be subjected to a complex pattern of regulation by coenzyme availability, product inhibition and ion effects (Giberti et al., 2014; Forlani et al., 2015a), supporting a so far underestimated role of P5CR in controlling stress-induced proline accumulation. These mechanisms may allow the plant enzyme to respond to wide fluctuations of P5C synthesis by either P5CS isozymes or OAT without the need of a transcriptional control. However, the molecular basis of the pyridine nucleotide coenzyme preference, i.e., the P5CR protein fragment responsible for NADH/NADPH discrimination, has not yet been determined. Also, the residues connected to the lack of feed-back inhibition by proline and those involved in inhibition or stimulation of the P5CR activity by chloride anions have not yet been described.

Here we report the high-resolution crystal structure of P5CR from *M. truncatula* (*Mt*), a model legume plant. For these studies, *Mt*P5CR was expressed heterologously in *Escherichia coli* cells, purified and characterized. Four crystal structures were then solved: unliganded *Mt*P5CR, its complexes with NAD^+^, NADP^+^, and L-proline. The structural data were related to the enzymatic properties, with special attention to the coenzyme preference (NADPH vs. NADH) and modulation of *Mt*P5CR activity by chloride anions. Additionally, because a 3-(*N*-morpholino)propansulfonate (MOPS) molecule was found within the active center of the *Mt*P5CR structure, the inhibitory properties of some sulfonates have been investigated, allowing us to propose 2-[4-(2-hydroxyethyl)piperazin-1-yl]ethanesulfonic acid (HEPES) as a putative starting molecule in the design of new P5CR inhibitors.

## Materials and methods

### Cloning, overexpression, and purification of *Mt*P5CR

Total RNA was isolated from *M. truncatula* leaves using the RNeasy Plant Mini Kit (Qiagen). SuperScript II reverse transcriptase (Life Technologies) with oligo dT (15 and 18) primers was utilized to obtain the complementary DNA (cDNA). The cDNA was used as a template to acquire the *MtP5CR* gene sequence by polymerase chain reaction. The primers used (Forward: TACTTCCAATCCAATGCCATGGAAATCATTCCGATCCCCGC, Reverse: TTATCCACTTCC AATGTTATCAGGAAAGCTCTTGGCTGCGTTTA) allowed for incorporating the gene into the pMCSG68 vector (Midwest Center for Structural Genomics Eschenfeldt et al., 2013) by ligase-independent cloning (Kim et al., 2011). The pMCSG68 vector introduces a His_6_-tag followed by the tobacco etch virus (TEV) protease cleavage site at the N-terminus of the expressed protein. The correctness of the insert was confirmed by DNA sequencing.

Overexpression was carried out in BL21 Gold *E. coli* cells (Agilent Technologies). The bacteria were cultured with shaking at 210 rpm in LB medium supplemented with 150 μg mL^−1^ ampicillin at 37°C until the OD_600_ reached 1.0. The temperature was lowered to 18°C and isopropyl-β-D-thiogalactopyranoside (IPTG) was added to a final concentration of 0.5 mM. The culture was grown for 18 h and then centrifuged at 3500 × *g* for 10 min at 4°C. A cell pellet from 1 L culture was resuspended in 35 mL of binding buffer (50 mM Tris-HCl pH 8.0, 500 mM NaCl, 20 mM imidazole, 1 mM tris(2-carboxyethyl)phosphine [TCEP]) and stored at −80°C. The samples were thawed and the cells were disrupted by sonication using four bursts for a total duration of 4 min, with 20 s intervals for cooling. Cell debris was pelleted by centrifugation at 25,000 × *g* for 30 min at 4°C. The supernatant was applied to a column packed with 6 mL of HisTrap HP resin (GE Healthcare), connected to a VacMan manifold (Promega) and the chromatographic process was accelerated with a vacuum pump. After binding, the column was washed with five 40-mL volumes of the binding buffer and the His_6_-tagged MtP5CR was eluted with 20 mL of elution buffer (50 mM Tris-HCl pH 8.0, 500 mM NaCl, 300 mM imidazole, 1 mM TCEP). The His_6_-tag was cleaved with TEV protease (final concentration 0.1 mg mL^−1^) and the excess imidazole was removed by dialysis (overnight at 4°C) simultaneously. The solution was mixed with HisTrap HP resin to eliminate the cleaved His_6_-tag and the His_6_-tagged TEV protease. The flow-through was collected, concentrated to 4 mL and applied onto a HiLoad Superdex 200 16/60 column (GE Healthcare) equilibrated with a buffer composed of 50 mM Tris-HCl pH 8.0, 200 mM NaCl, and 1 mM TCEP. Size exclusion chromatography yielded a homogenous protein fraction.

### Crystallization and data collection

The *Mt*P5CR solution was concentrated using Amicon concentrators (Millipore) to 22 mg mL^−1^, as determined by measuring the absorbance at 280 nm on the basis of an extinction coefficient of 17,000 M^−1^ cm^−1^. Crystallization screening was performed using a robotic sitting drop vapor diffusion setup (Mosquito). The most promising hits were optimized manually in hanging drops. The final conditions were obtained from the Morpheus screen (Molecular Dimensions) A7 solution (100 mM HEPES/MOPS buffer pH 7.5, 10% polyethylene glycol 4000, 20% glycerol, 30 mM MgCl_2_, 30 mM CaCl_2_). The crystallization drop was composed of 4 μL of protein and 2 μL of the reservoir solution. Crystals with ~0.5 × 0.5 × 0.5 mm dimensions appeared after 5 days at 19°C. Aiming at obtaining *Mt*P5CR/NAD^+^ and *Mt*P5CR/NADP^+^ complexes, the protein was incubated with either of the two coenzymes (at a 4 mM final concentration) for 10 min at room temperature and centrifuged at 17,000 × *g* prior to the crystallization setup. The analogous cocrystallization failed while obtaining the structure of the L-proline complex, as determined by inspection of the resulting electron density maps. Therefore, 0.1 μL of 1 M L-proline, dissolved in the reservoir solution, was added to the drop containing grown crystals of the unliganded *Mt*P5CR. The crystallization conditions provided sufficient cryo-protection and the crystals were harvested and flash frozen in liquid nitrogen.

The diffraction data collected at beamline 19-ID at Advanced Photon Source, Argonne, USA (Rosenbaum et al., 2006). The diffraction images for the unliganded, NAD- and NADP-bound *Mt*P5CR were processed with *XDS* (Kabsch, 2010), while the *HKL3000* package (Minor et al., 2006) was utilized in the case of the *Mt*P5CR/L-proline complex. The data collection and processing statistics are summarized in Table 1. Diffraction data at 1.7196 Å wavelength were collected at beamline 19-BM. To increase the multiplicity of measurements, four datasets, each differing only by 10-pixels shifts of the detector, were processed and scaled with *XDS*.

**Table 1:** **Data-collection and refinement statistics; values in parentheses correspond to the highest resolution shell**.

**MtP5CR:**	**Unliganded**	**NAD^+^**	**NADP^+^**	**L-Proline**	**Anomalous**
**DATA COLLECTION**					
Wavelength (Å)	0.9790	0.9790	0.9786	0.9786	1.7196
Temperature (K)	100	100	100	100	100
Space group	*P*1	*P*1	*P*1	*P*1	*P*1
Unit cell parameters					
*a, b, c* (Å)	86.8, 100.6, 101.2	87.5, 100.8, 101.6	87.2, 100.3, 100.8	87.2, 101.1, 101.5	87.2, 101.2, 101.9
α, β, γ (°)	67.9, 85.3, 89.3	67.5, 85.9, 89.8	68.1, 85.8, 89.3	67.4, 85.4, 89.6	67.7, 85.6, 89.5
Oscillation range (°)	0.5	0.5	1	0.5	1
No. of images	680	680	300	500	4 × 240
Resolution (Å)	40.0-1.70 (1.74-1.70)	40.0-1.85 (1.90-1.85)	40.0-1.95 (2.06-1.95)	40-2.10 (2.14-2.10)	40.0-2.50 (2.57-2.50)
Reflections collected/Unique	1383055/338502	978057/266309	732206/225659	481562/183949	1097686/208539
Completeness (%)	97.2 (95.6)	97.4 (96.2)	96.9 (92.5)	97.7 (96.7)	93.8 (83.8)
Multiplicity	4.1 (3.4)	3.7 (3.7)	3.2 (3.2)	2.6 (2.6)	5.3 (4.9)
*R*_meas_^a^(%)	5.0 (79.9)	5.3 (75.0)	13.6 (87.6)	8.6 (60.8)	4.2 (22.9)
<*I*/σ(*I*)>	15.2 (1.9)	17.7 (2.0)	10.7 (2.0)	13.3 (1.9)	33.4 (7.3)
**REFINEMENT**					
R_free_ reflections	2708	2131	2257	1941	
No. of atoms (non-H)	21823	22411	22670	20686	
Protein	20277	20161	20086	19911	
Ligands	75	580	620	80	
Solvent	1471	1670	1964	695	
*R*_work_/*R*_free_ (%)	16.0/18.0	15.5/17.7	15.5/17.8	18.0/19.7	
Mean atomic displacement parameter (Å^2^)	52.7	41.4	31.3	74.0	
RMSD from ideal geometry					
Bond lengths (Å)	0.020	0.021	0.019	0.017	
Bond angles (°)	1.9	1.9	1.8	1.6	
Ramachandran statistics (%)					
Favored	99.5	99.1	99.0	99.3	
ALLOWED	0.5	0.9	1.0	0.7	
Outliers	0.0	0.0	0.0	0.0	
PDB code	5bse	5bsf	5bsg	5bsh	

a *R_meas_ = redundancy independent R-factor (Diederichs and Karplus, 1997)*.

### Determination and refinement of the crystal structures

The crystal structure of *Mt*P5CR was determined by molecular replacement using the *PHASER* program (McCoy et al., 2007). A decamer created from the closest homolog with the available crystal structure, namely the human P5CR with 34% identity (PDB ID: 2izz), was used as a search probe. Automatic model building was carried out with the online version of *ARP/wARP* (Langer et al., 2008). The atomic coordinates were placed inside the unit cell using the *ACHESYM* server (Kowiel et al., 2014). The protein chain from the unliganded *Mt*P5CR (the structure without a bound coenzyme or L-proline) was used as the initial model for refinement of the other complexes, which are all isomorphous. *COOT* (Emsley et al., 2010) was used for manual fitting in the electron density maps between rounds of model refinement using Refmac (Murshudov et al., 2011) with local non-crystallographic symmetry (NCS) restraints. Additional, secondary structure restraints, generated with ProSMART (Nicholls et al., 2014), were applied for poorly defined regions of the G chains of the unliganded *Mt*P5CR and the complex with L-proline. Translation/Libration/Screw (TLS) parameters (Winn et al., 2001) were refined for twenty groups in each of the four structures. More precisely, each protein chain was divided into two TLS groups corresponding to the two distinctive domains: the NAD(P)-binding domain (N-terminus:Asp169) and the dimerization domain (Lys170:C-terminus). Riding hydrogen atoms for the protein chain were included in the refinement. The refinement statistics are listed in Table 1.

### Biochemical analysis

The physiological, forward reaction of P5CR was measured at 35°C following the P5C-dependent oxidation of NAD(P)H. Unless otherwise specified, the assay mixture contained 50 mM Tris-HCl buffer pH 7.5, 1 mM NADH or 0.5 mM NADPH, and 2 mM DL-P5C (equivalent to 1 mM L-P5C; Williams and Frank, 1975) in the final volume of 0.2 mL. DL-P5C was synthesized by the periodate oxidation of δ-allo-hydroxylysine (Sigma H0377) and purified by cation-exchange chromatography, as described previously (Forlani et al., 1997). P5C solution was neutralized with 1 M Tris base (pH ~11) immediately before use. A limiting amount of *Mt*P5CR (7 ng of the purified protein in 10 μL buffer, 1.3 nM final concentration) was added to the pre-warmed mixture. The decrease of absorbance at 340 nm was recorded with 30 s intervals for 5 min through an optical path of 0.5 cm. Activity was calculated from the initial linear rate on the assumption of a molar extinction coefficient for NAD(P)H of 6220 M^−1^ cm^−1^. Linear regression analysis was computed with Prism 6 (version 6.03, GraphPad Software, Inc., USA).

To calculate apparent Michaelis-Menten constants (K_M(app)_), invariable substrates were fixed at the same levels as in the standard assay. The concentration of L-P5C ranged from 50 to 400 μM with NADH as the electron donor, and from 25 to 200 μM with NADPH as the electron donor. The concentrations of NADH and NADPH ranged from 200 to 800 μM and from 50 to 200 μM, respectively. When evaluating the effect of NaCl, the L-P5C concentration was reduced to 200 μM to minimize the carryover of chloride anions. All assays were performed in triplicate. K_M_ and V_max_ values, as well as the concentrations causing 50% inhibition (IC_50_) of P5CR activity, K_I_ values and their confidence intervals were estimated by non-linear regression analysis using Prism 6. Catalytic constants were calculated from V_max_ values, taking into account a homodecameric composition of the native holoenzyme, each monomer having a molecular mass of 28.5 kDa.

### Other software used

Molecular figures were created with UCSF *Chimera* (Pettersen et al., 2004). RMSDs were calculated with the same program for Cα atoms within 3 Å radius. Assignment of secondary structure elements was based on the DSSP algorithm (Kabsch and Sander, 1983) and PDBsum web server (de Beer et al., 2014). Ramachandran plots were calculated in *Rampage* (Lovell et al., 2003).

## Results

### Cloning of the gene coding for pyrroline-5-carboxylate reductase in *M. truncatula*

**Figure 2:**
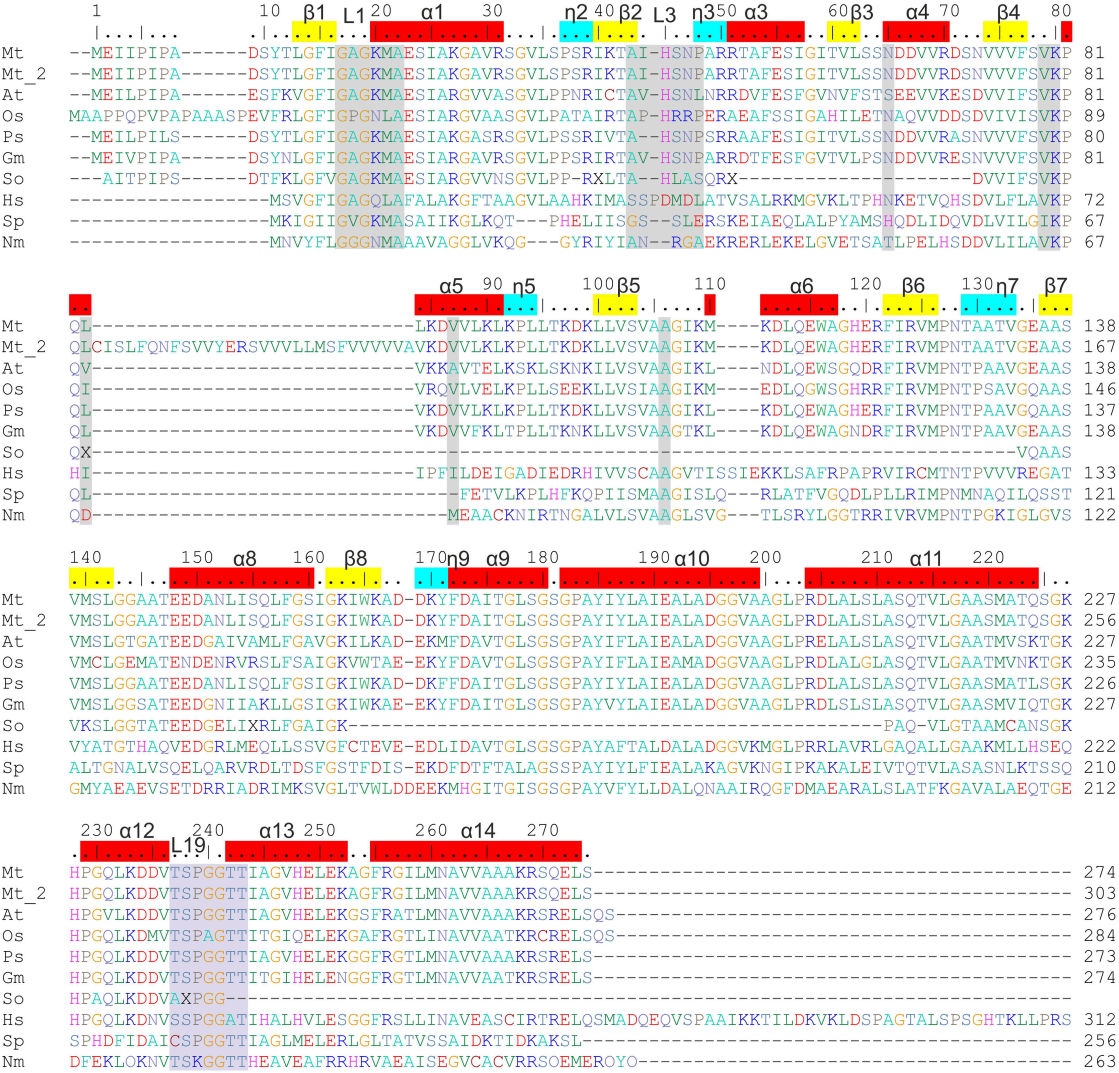
**Sequence alignment of P5CRs from various sources**. The following organisms are listed and square brackets indicate UniProt accession numbers: *Mt, Medicago truncatula* [G7KRM5], Mt_2 indicates the second isoform of *Mt*P5CR [A2Q2Y7]; *At, Arabidopsis thaliana* [P54904]; *Os, Oryza sativa* [Q8GT01]; *Ps, Pisum sativum* [Q04708]; *Gm, Glycine max* [K7KEQ2]; *So, Spinacia oleracea*, only partial sequence is available (Murahama et al., 2001); *Hs, Homo sapiens* [P32322]; *Sp, Streptococcus pyogenes* [Q9A1S9]; *Nm, Neisseria meningitides* [Q9K1N1]. Amino acid residues are colored according to the type of residue. Numbering on the top reflects the *Mt*P5CR sequence, while that on the right of each row is protein-specific. Residues interacting with NAD(P)^+^ and L-proline in *Mt*P5CR structure and conserved among other species are highlighted in gray and light blue, respectively. α-Helices, 3_10_ helices and β-strands corresponding to *Mt*P5CR structure are depicted on top as red, blue and yellow bars, respectively. L1, L3, and L19 indicate the location of loops 1, 3, and 19, which were discussed in the paper. Seven C-terminal residues from the human P5CR sequence were omitted. The human and the two bacterial sequences were chosen for alignment as their crystal structures had already been determined.

According to the UniProt knowledgebase (UniProt Consortium, 2015), in the legume *M. truncatula* there should be two P5CR isoforms (*Mt*P5CR_1 and *Mt*P5CR_2). However, they have a nearly identical sequence, except for a 29 amino acid insert in *Mt*P5CR_2 between Leu83 and Leu84 of *Mt*P5CR_1 (Figure 2). This insert contains an unusually high number of hydrophobic residues, and a BLAST (Altschul et al., 1997) search revealed no P5CRs with similar inserts in other species. Moreover, a recent and comprehensive analysis of plant P5CRs from a phylogenetic perspective failed to find support for the occurrence of two genes in this species (Forlani et al., 2015b). In another legume, *Glycine max*, two genes are indeed present that show 94% identity, suggesting recent duplication, but none of them contains an insert similar to that of *Mt*P5CR_2. Therefore, *Mt*P5CR_1 was cloned as the genuine gene coding for P5CR in *M. truncatula*. The sequence conservation between *Mt*P5CR and its orthologs (Figure 2) is high among plants, and exceptionally high among legumes, equaling (identity/similarity in %) 73/87 for *Arabidopsis thaliana* (*At*), 63/77 for *Oryza sativa* (*Os*), 92/95 for *Pisum sativum* (*Ps*), 85/93 for *Glycine max* (*Gm*), 34/51 for *Homo sapiens* (*Hs*), 30/50 for *Streptococcus pyogenes* (*Sp*), and 29/46 for *Neisseria meningitides* (*Nm*).

### *Mt*P5CR shows a complex pattern of activity regulation by substrates, products, and salts

*Mt*P5CR kinetic parameters were determined. Results are summarized in Table 2. Maximal activity was obtained with NADH as the electron donor, corresponding to a catalytic constant of almost 3000 catalytic events s^−1^ *per* monomer. When NADPH was used as the coenzyme instead of NADH, a remarkably lower V_max_ value was obtained. However, the K_M(app)_ found for P5C is 12-fold lower when NADPH is used as the coenzyme. The corresponding constants for either coenzyme showed a similar trend, with apparent K_M_ values of 39 and 468 μM for NADPH and NADH, respectively. Fully saturating conditions for NADH were not obtained, even at the highest concentration used (1 mM).

**Table 2:** **Kinetic features of ***Medicago truncatula*** P5CR**.

K_M(app)_ L-P5C _(NADH)_	240 ± 24 μM
K_M(app)_ L-P5C _(NADPH)_	20 ± 2 μM
K_M(app)_ NADH	468 ± 22 μM
K_M(app)_ NADPH	39 ± 3 μM
V_max_ L-P5C _(NADH)_	97.8 ± 4.7 μkat (mg protein)^−1^
V_max_ L-P5C _(NADPH)_	35.1 ± 0.8 μkat (mg protein)^−1^
V_max_ NADH	92.4 ± 2.1 μkat (mg protein)^−1^
V_max_ NADPH	36.1 ± 0.7 μkat (mg protein)^−1^
kcat _(NADH)_	2972 s^−1^
kcat _(NADPH)_	1113 s^−1^
kcat/K_M__(NADH)_	6.35 × 10^6^ M^−1^ s^−1^
kcat/K_M__(NADPH)_	2.85 × 10^7^ M^−1^ s^−1^

**Figure 3:**
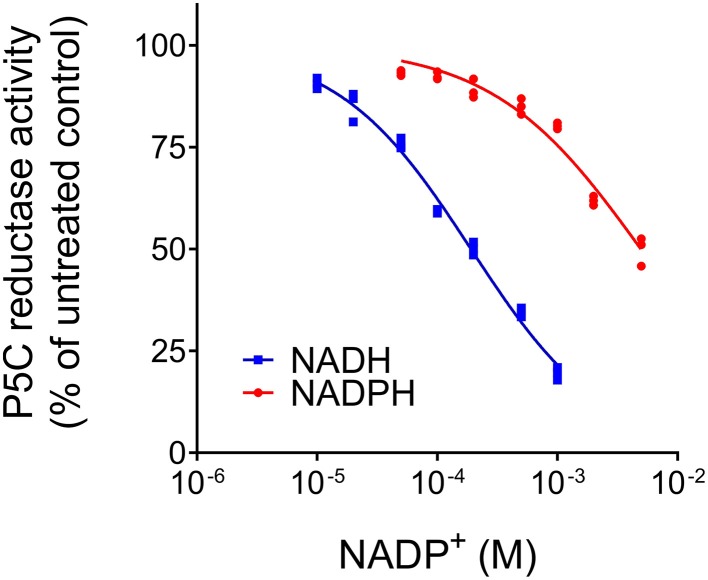
**Effect of NADP^+^ on the activity of ***Mt***P5CR**. The standard reaction mixture, containing 1 mM NADH or 0.5 mM NADPH, was added with increasing concentrations of the oxidized dinucleotide. The resulting activity was expressed as a percent of mean value in untreated controls. Three replications were carried out for each treatment. Non- linear regression analysis of data [log(inhibitor) vs. normalized response-Variable slope] was computed using Prism 6 for Windows, version 6.03.

When the activity of *Mt*P5CR (NADH vs. NADPH-dependent reaction) was measured in the presence of increasing concentrations of NADP^+^ or NAD^+^, an inhibitory effect was found only in the case of NADP^+^ (Figure 3). NAD^+^ was ineffective in the range of concentrations tested (up to 10 mM, data not shown). Interestingly, the inhibition caused by NADP^+^ was drastically stronger when NADH was the electron donor. With equimolar levels of the oxidized and the reduced forms, the NADH-dependent activity was inhibited by 80%, whereas the NADPH-dependent reaction rate was reduced only by 20%. Quite unexpectedly, and contrary to what previously found for the enzyme from *A. thaliana* and rice (Giberti et al., 2014; Forlani et al., 2015a), proline in the range from 1 to 100 mM inhibited neither the NADH-dependent nor the NADPH-dependent activity of *Mt*P5CR.

**Figure 4:**
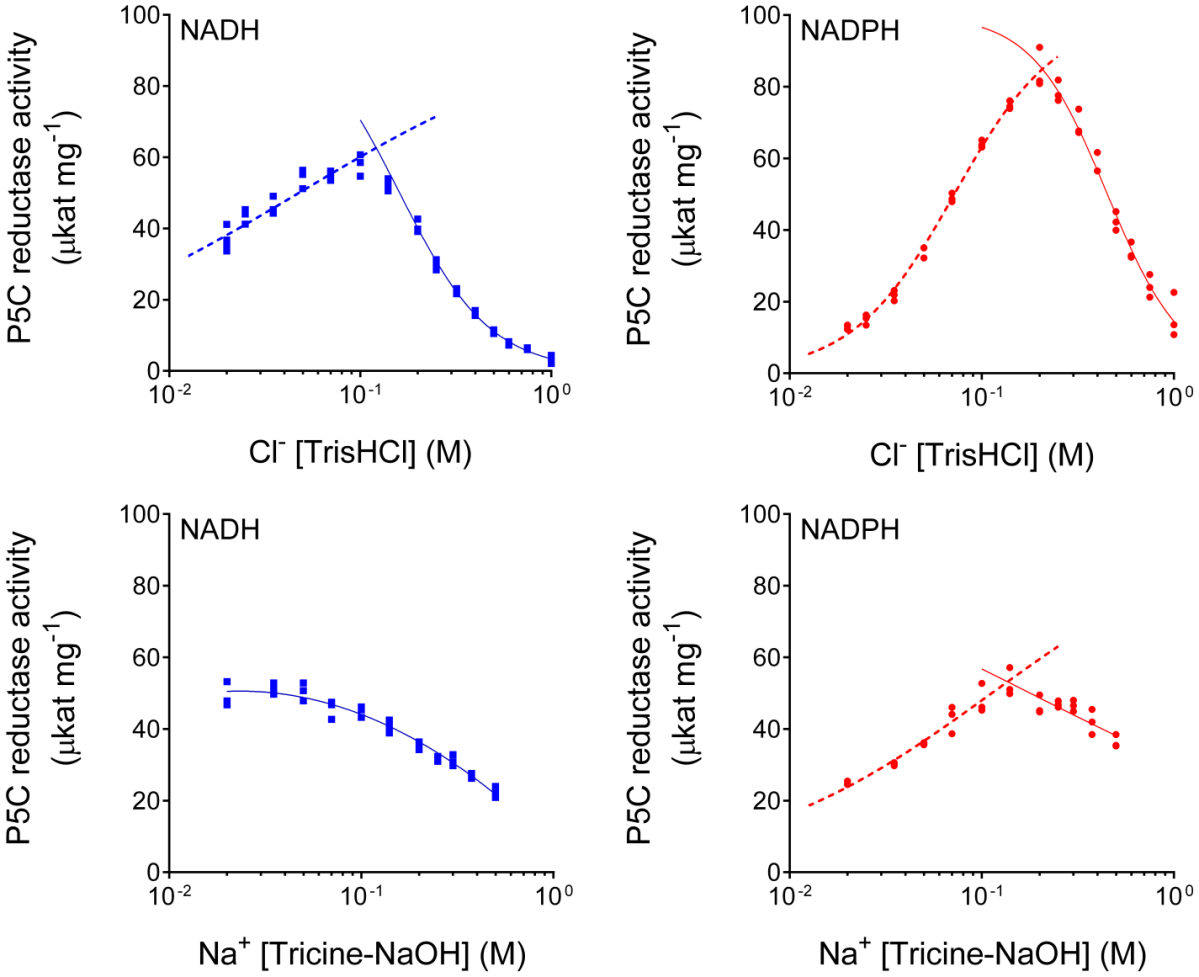
**Differential effect of Cl^−^ anions and Na^+^ cations on ***Mt***P5CR activity**. To reduce the carryover of ions in the standard reaction mixture, the enzyme was assayed in the presence of 0.2 mM L-P5C (in 20 mM Tris-HCl buffer pH 7.5) with either 1 mM NADH or 0.5 mM NADPH as the electron donor. To avoid unspecific effects due to pH variations, after fixing the desired concentration of Cl^−^ and Na^+^ ions, the pH was brought to pH 7.75 with Tris base or tricine, respectively. Three replications were carried out for each treatment. Non-linear regression analysis of data [log(inhibitor) vs. normalized response-Variable slope for enzyme inhibition; log(agonist) vs. normalized response-Variable slope for enzyme stimulation] was computed using Prism 6 for Windows, version 6.03.

When the impact of chloride ions on enzyme activity was evaluated, results were contrasting depending on the concentration. In the range of 20–200 mM, a progressive stimulation was observed. Over this threshold, the results were opposite, as the activity was steadily reduced (data not shown). Also in this case, the effect was different if NADH or NADPH was the coenzyme, with IC_50_ values of 485 ± 21 or 704 ± 7 mM, respectively. However, with NaCl it is impossible to distinguish the effect of Cl^−^ anions from that of Na^+^ cations. Therefore, the experiment was repeated by adding the two ions separately, as Na-Tricine or Tris-HCl. Preliminary trials ruled out the possibility that tricine anions or Tris cations significantly influenced enzyme activity (data not shown). The results, shown in Figure 4, clearly pointed at chlorides as the main effectors. Once again, the effect differed depending on the coenzyme used. With NADH, a 1.8-fold stimulation of enzyme activity was found with the addition of 100 mM Cl^−^ to the reaction mixture, then activity was progressively inhibited, with an IC_50_ value of 161 ± 6 mM. Under the same conditions, but with NADPH instead of NADH, P5CR activity was stimulated seven-fold, and the subsequent inhibition took place at higher concentrations, with an IC_50_ value of 451 ± 15 mM. The addition of equimolar levels of Na^+^ had, on the contrary, minor effects and, in the case of NADH, only a progressive inhibition of enzyme activity was evident.

### The *Mt*P5CR protein is a decamer of five dimers with higher structural similarity with the bacterial orthologs than with the human enzyme

Crystals of unliganded *Mt*P5CR and its complexes with the products NAD^+^, NADP^+^ and L-proline were obtained, and their structures were refined using high-resolution diffraction data (Table 1). In this paper, the term “unliganded” refers to the structure without a coenzyme or L-proline, despite the fact that it actually contains non-protein and non-solvent ligands. The protein crystallized in the *P*1 space group, with one decamer (chains A–J) as the asymmetric unit. The decameric quaternary structure in the crystal lattice was in agreement with the size-exclusion elution profile of *Mt*P5CR (data not shown). Crystals of *Mt*P5CR contained about 57% solvent. Two to six (chain-dependent) N-terminal residues were not defined in the electron density maps and are not present in the deposited coordinate files. On the other hand, each C-terminus was modeled completely. In the complex with L-proline (2.1 Å resolution), the protein chain for residues 43–46 (in chains C and D) and 43–49 (chain G) was disordered. The structures with a bound coenzyme, despite having poorer data resolution (1.85 Å with NAD^+^ and 1.95 Å with NADP^+^) than the unliganded structure (1.7 Å), had lower average atomic displacement parameters, and allowed for tracing more water molecules (Table 1).

**Figure 5:**
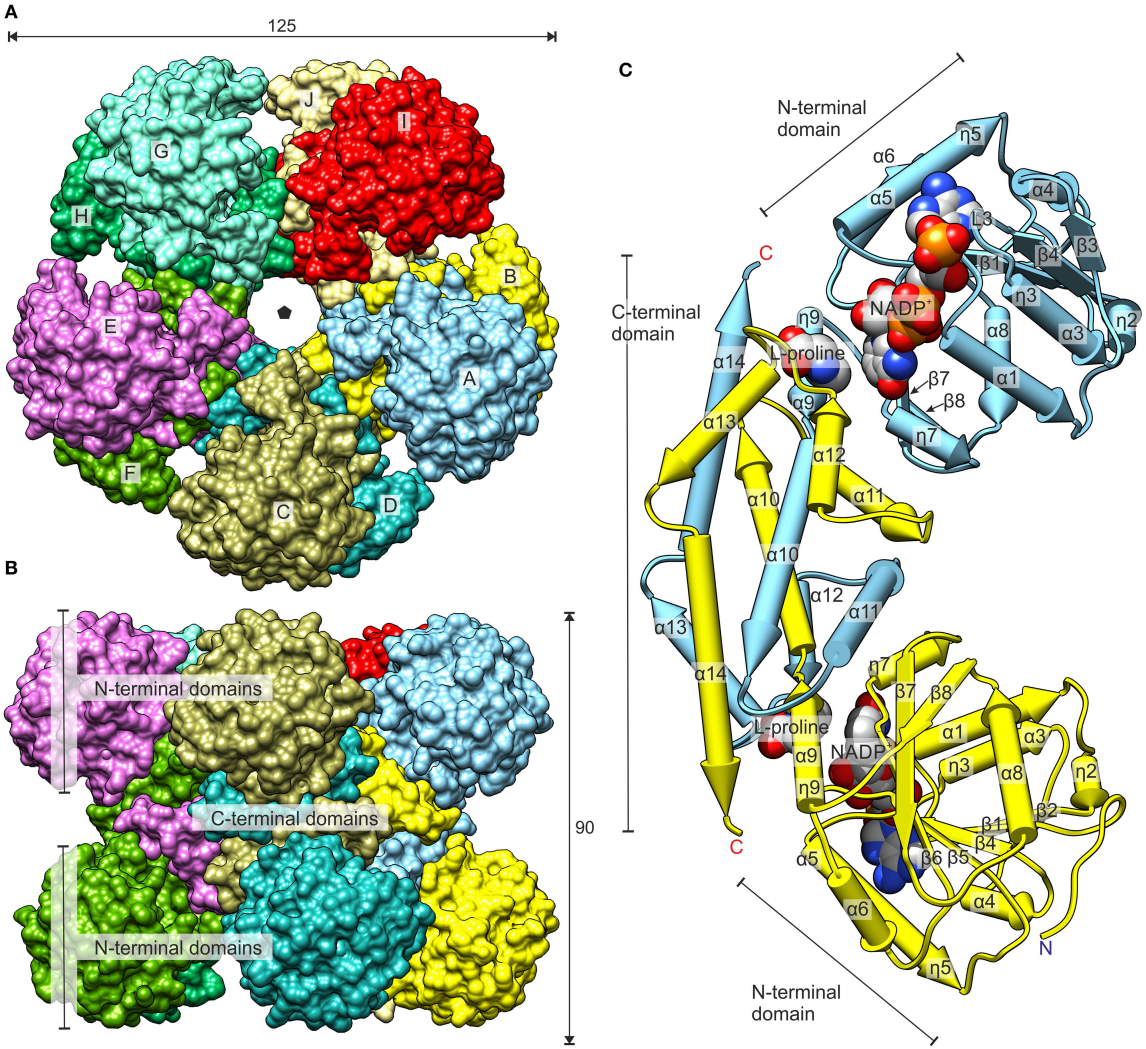
**Overall structure of ***Mt***P5CR**. **(A)** The top view. The black pentagon indicates the five-fold NCS axis. **(B)** The side view. Dimensions are given in Å. **(C)** The dimer of *Mt*P5CR. The structures with NADP^+^ and with L-proline are superimposed (RMSD 0.34 Å) to show the binding sites of the coenzyme and product. The protein chain from the L-proline complex as well as MOPS and Cl^−^ from the NADP^+^ structure are omitted for clarity. Chains A and B are shown.

The donut-shaped decamer of *Mt*P5CR can be described as a pentamer of symmetric, bean-like dimers (Figure 5) that are related to each other by a five-fold NCS axis. The molecules are organized in such way that the N-terminal domains point outwards of the hollow core of the decamer. As a result, a large groove around the decamer is formed in a plane perpendicular to the five-fold NCS axis. Each N-terminal domain is an α/β/α sandwich of a nucleotide-binding Rossmann fold composed of eight helices wrapped around an eight-stranded β-sheet. The organization of strands within the β sheet is as follows: β3-β2-β1-β4-β5-β6-β7^*^-β8^*^ (where an asterisk indicates the opposite direction of the strand). Between the strands β6 and β7^*^, a β-hairpin is formed that contains a 3_10_ helix (η7). There are four fragments within the N-terminal domain that have a 3_10_ helix twist (η2, η3, η5, η7; numbering of the helices is consecutive, regardless of their type).

The C-terminal domain of *Mt*P5CR consists of six helices and includes a hinge that probably allows for independent movement of the N-terminal domains in solution. This hinge results in a triple deformation of the ninth helix: (i) a tight 3_10_ helix (η9), (ii) backbone torsion angles of Leu178 outside the expected range, and (iii) a break between the α9 and α10 helices at Gly180. The C-terminal domains are responsible for incorporating two monomers into a dimer. More precisely, five C-terminal helices (α10–α14) are swapped between the monomers within the dyad and form a multi-helix bundle. The surface areas of isolated monomers and dimers equal 12,380 and 19,560 Å^2^, respectively. Hence, the calculated buried surface area upon dimer formation is extensive, equaling 5200 Å^2^. Each C-terminal domain also contains the P5C binding site that is the reduction reaction venue.

**Figure 6:**
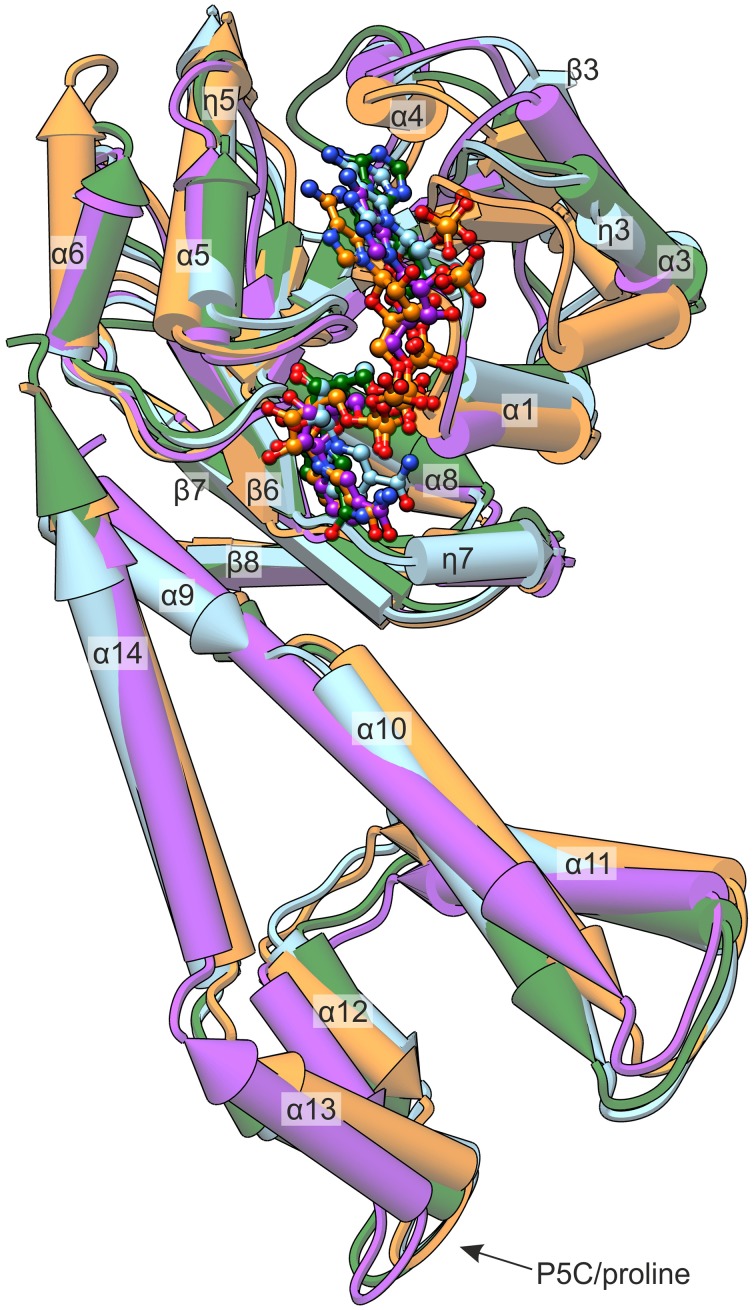
**Comparison of known P5CR crystal structures**. The presented enzymes originate from: *Mt*P5CR/NADP^+^ complex (this work, light blue); *Hs*P5CR/NAD^+^ (PDB ID: 2izz, orange); *Nm*P5CR/NADP^+^ (2ag8, green); and *Sp*P5CR/NADP^+^ (2ahr, purple). The protein molecules are shown as pipe-and-plank models, while the coenzymes are presented as ball-and-stick models. Secondary structure elements are denoted as: α, α-helices; η, 3_10_ helices; β, β-strands.

*Mt*P5CR, like other plant P5CRs, shares a higher sequence identity with the human ortholog than with the bacterial ones (Forlani et al., 2015b). Interestingly, from the structural alignment (Figure 6) it appears that *Mt*P5CR is actually more similar to *Sp*P5CR [PDB ID: 2ahr, (Nocek et al., 2005); root-mean-square deviation (RMSD) 1.6 Å] and *Nm*P5CR (2ag8, (Nocek et al., 2005); RMSD 1.5 Å) than to human P5CR (2izz, Pike et al., unpublished, RMSD 1.9 Å).

Most of the differences are noticeable within the N-terminal domain and apply to the helices. More precisely, the η3 and the α6 helices that in *Mt*P5CR are similar to bacterial orthologs have different inclinations in the human protein. On the other hand, the 5th helix of the *Mt*P5CR enzyme is longer (by the η5 fragment) than the corresponding helices in the two prokaryotic P5CRs, and resembles the corresponding helix of the human ortholog. The NAD(P)^+^ is bound in each of the four compared structures in a similar way (Figure 6) and, when the protein chains are superposed, the corresponding atoms of the coenzymes deviate by less than 3.5 Å. The major difference concerning the C-terminal domain is the break between helices α9 and α10, which is observed in the *Mt, Hs* and *Nm* enzymes, but is absent in the *Sp* ortholog. *Nm*P5CR is a dimer in solution (Nocek et al., 2005), while *Sp, Hs*, and *Mt* enzymes are decamers. Therefore, the break between helices α9 and α10 is unrelated to oligomerization.

### Pyridine dinucleotide binding is consistent with the preference for NADPH over NADH as the electron donor

The catalytic centers of P5CRs are located between the dinucleotide-binding domain (N-terminal) and the dimerization domain (C-terminal) (Nocek et al., 2005). However, the substrates and the coenzymes are delivered to the catalytic centers by different protein subunits that form the dimer. In other words, P5C bound by the C-terminal domain of protein subunit A is reduced by NAD(P)H brought by the N-terminal domain of its dimer mate, subunit B. The reaction can occur in the second active site within the dimer simultaneously, namely the hydride provided by NAD(P)H bound by subunit A can reduce P5C to L-proline in the active center of subunit B.

**Figure 7:**
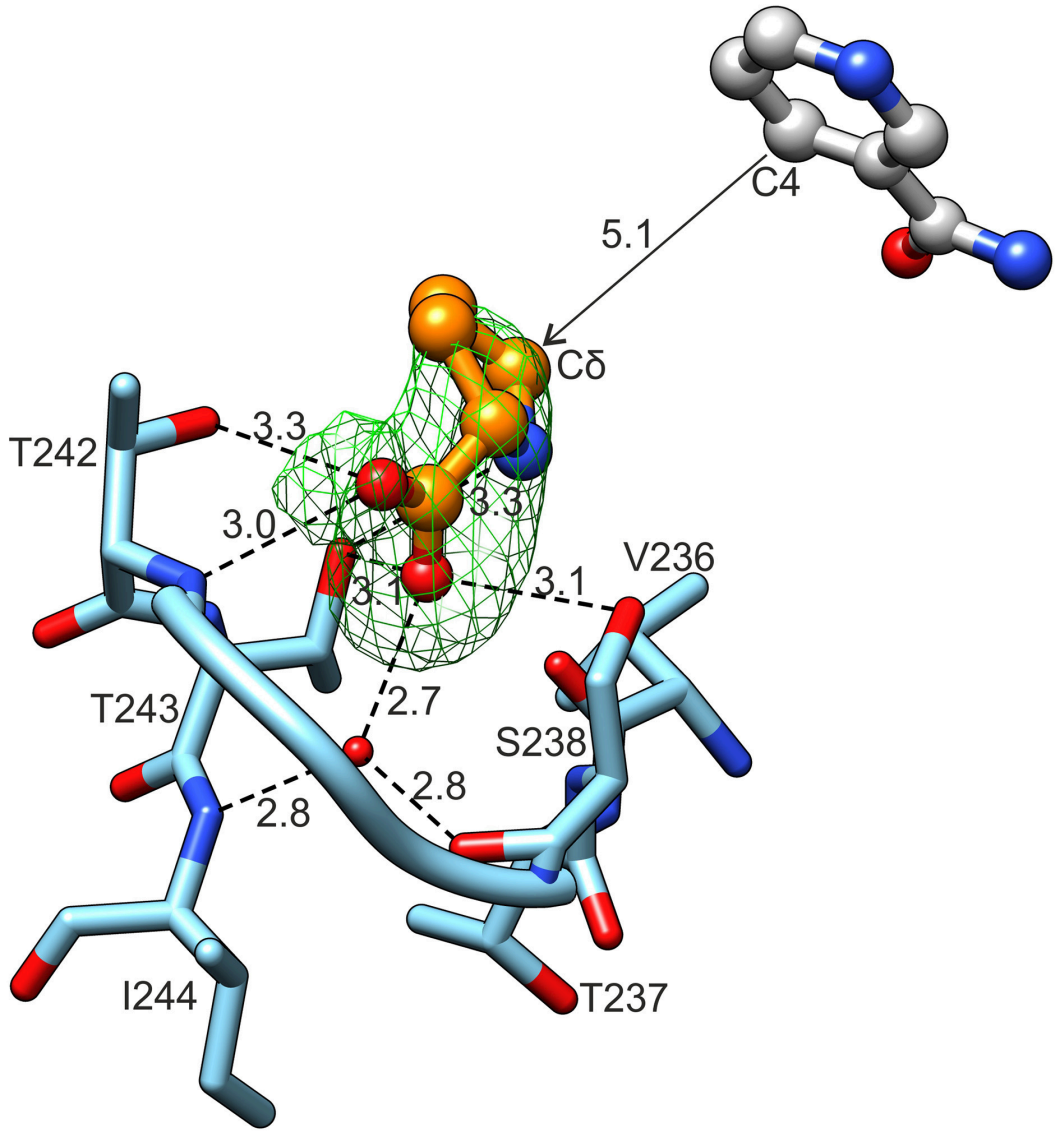
**L-Proline binding**. Green mesh represents omit difference *F*_*o*_*-F*_*c*_ electron density map contoured at the 4 σ level. Nicotinamide originates from the superposed chain B of the *Mt*P5CR/NADP^+^ complex. Distances are given in Å.

Close examination revealed an electron density matching of L-proline surrounded by the L19 loop, between Ser238 and Thr242 in each of the 10 chains of the *Mt*P5CR/L-proline complex (Figure 7). The reaction product interacts with the protein chain through both carboxyl O atoms. The O1 atom, at an *anti* position with respect to N of L-proline, forms hydrogen bonds with the backbone N of Thr242 and Oγ of the same residue. The O2 atom (*syn* to N of L-Pro) interacts with Oγ of Ser238, Oγ of Thr243 and a well-ordered water molecule at the bottom of the cavity. This water molecule is hydrogen-bonded to the carbonyl O of Ser238 and the backbone N of Ile244. The N atom of proline participates in product binding through formation of a weak hydrogen bond with Oγ of Thr243.

**Figure 8:**
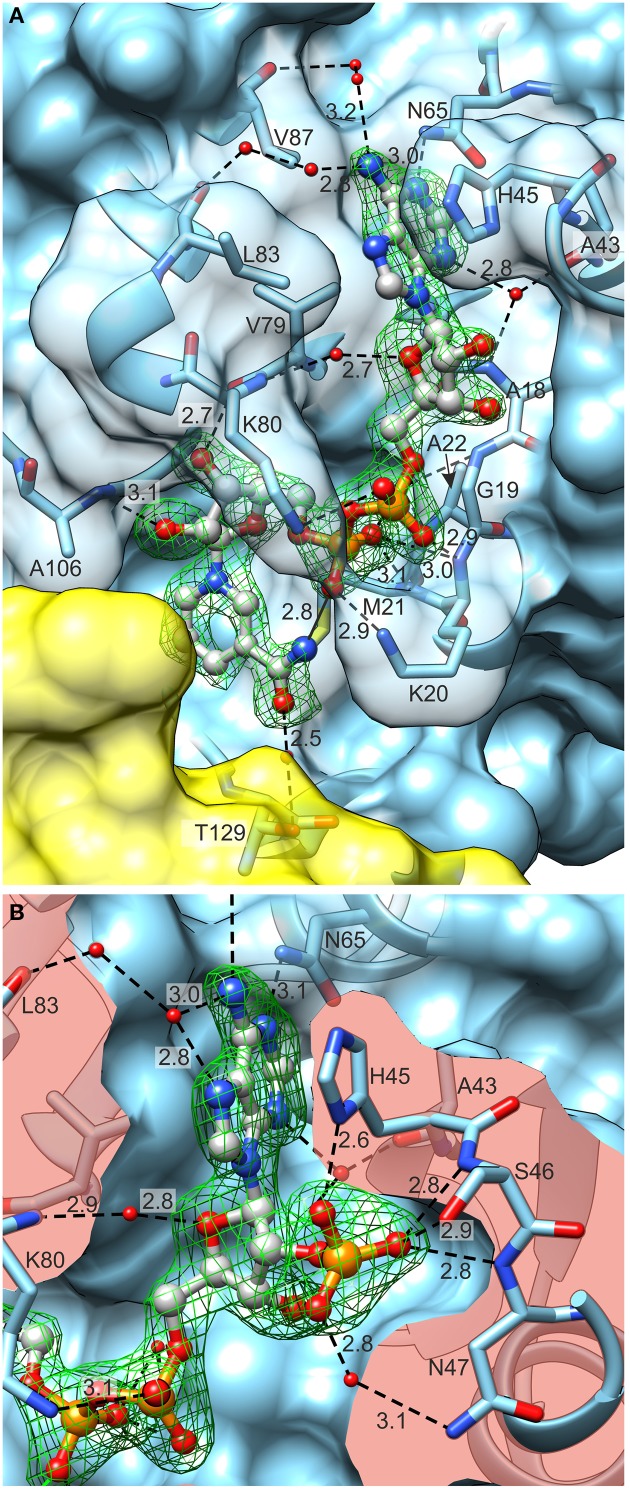
**Coenzyme (ball-and-stick model) binding by ***Mt***P5CR**. **(A)** The *Mt*P5CR/NAD^+^ complex. **(B)** The close-up view of the ribose 2′O-bound phosphate in NADP^+^. Both figures are oriented as in Figure 1C. Green mesh areas represent omit difference *F*_*o*_–*F*_*c*_ electron density maps contoured at the 4 σ level. Water molecules that take part in binding interactions are depicted as small red balls. Chain B is represented as a yellow semitransparent surface. Note that Thr129 belongs to chain A and is only covered by a part of the C-terminal domain of chain B. The surface of interacting amino acids is semitransparent in **(A)** and clipped in **(B)** so it does not obscure the coenzymes. Black dashed lines represent bonding protein-coenzyme interactions. Intramolecular interaction between the N atom of nicotinamide and O from pyrophosphate group of NAD^+^ is depicted as a black solid line at the bottom of **(A)**. Distances are given in Å.

Concerning hydride donors, the complexes with NAD^+^ and NADP^+^ showed that both coenzymes are bound by the N-terminal domain of *Mt*P5CR *via* multiple hydrogen bonds (Figure 8) created mostly by the backbone N and O atoms of the protein. The coenzyme-binding groove contains the dinucleotide-binding motif of the Rossmann fold, which, in the case of *Mt*P5CR, encompasses residues _17_GAGKMA_22_, which correspond to the L1 loop and the N-terminal part of α1 helix.

In the case of NAD^+^ (Figure 8A), the adenine N1 atom interacts with Nδ of Asn65. The N3 atom of adenine forms water-mediated hydrogen bonds with the backbone N and O of Ala18 and Ala43, respectively. The binding of the N6 atom by the backbone O atoms of Leu83 and Val87 occurs *via* two solvent molecules in both cases. Additionally, adenine interacts by π–stacking to His45 with an interplanar distance of about 3.6 Å and an inclination below 6°. The adenine-adjacent ribose interacts *via* a single water-mediated hydrogen bond between the O atom of the monosaccharide and the backbone N atom of Lys80. The O atoms of the pyrophosphate group form strong ionic hydrogen bonds to the peptide amides of Lys20, Met21 and, *via* H_2_O, to Gly19 and Ala22. The overall dipole moment of helix α1, of which Lys20, Met21, and Ala22 are the N-terminal residues, also contributes to the increased electrostatic attraction. Additionally, the pyrophosphate interacts with the Nζ atoms of Lys20 and Lys80. These Nζ atoms are not well-defined in the electron density maps; they may, perhaps, bind with similar energies to various O atoms of pyrophosphate. These fully exposed Lys20 and Lys80 residues may act like claws that capture the coenzyme by its pyrophosphate moiety and seize the coenzyme from the solvent/cytoplasm at the initial stage of binding. These two residues are highly conserved in different species (Figure 2). The nicotinamide-adjacent ribose forms two direct hydrogen bonds between O2′ and the backbone NH of Ala106, as well as between O3′ and the carbonyl of Val79. The nicotinamide interacts *via* a water molecule with Oγ of Thr129. In addition, an intramolecular hydrogen bond between the amide N atom of nicotinamide and one of the pyrophosphate oxygens allows for determination of the correct orientation of the amide group. The *si*-side of the C4 carbon atom of the nicotinamide faces toward the catalytic center. This means that during the reduction reaction, the hydride would be transferred from the *pro*-S position of NAD(P)H (Figure 7).

NADP^+^ is bound in a very similar manner to NAD^+^ (Figure 8B), though differences arise from the presence of the additional phosphate group bound to O2′ of the adenine-adjacent ribose. This phosphate group causes rearrangement of the L3 loop (_44_IHSNP_48_) and contributes to a higher number of binding interactions. The three oxygen atoms of the phosphate group create ionic hydrogen bonds with: (i) the backbone N atoms of Ser46 and Asn47 and Oγ of Ser46 (O1 of phosphate), (ii) the Nδ atom of His45 (O2), and (iii) the Nδ atom of Asn47 (O3). One additional interaction occurs between the N7 atom of the adenine moiety and the water molecule that ultimately (by another water molecule) binds to carbonyl O of Leu83.

### Anions but not cations are present in the active site of *Mt*P5CR

**Figure 9:**
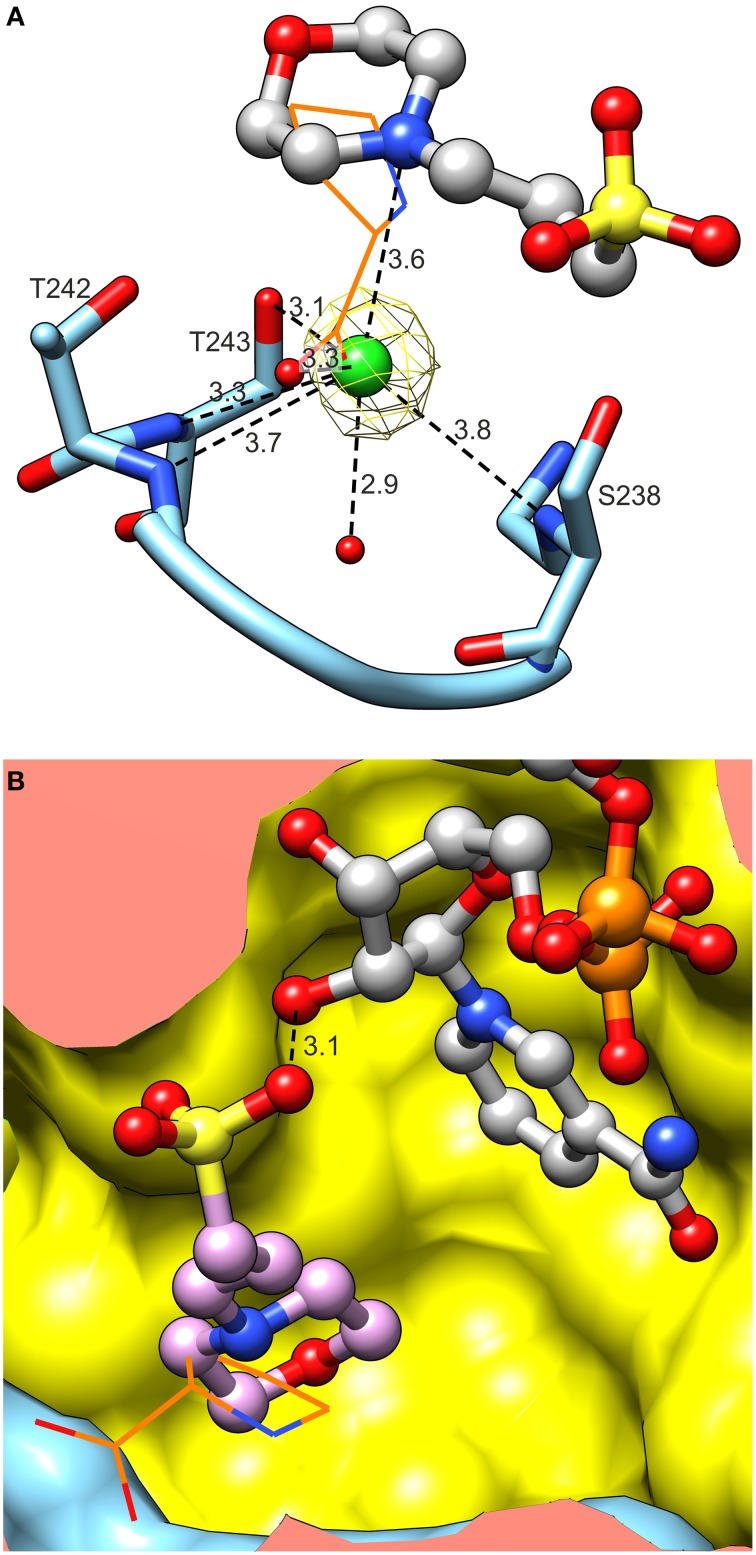
**Binding of activity modulators**. **(A)** Binding of chloride in the active site. Note multiple hydrogen bonds between Cl^−^ and peptide amides. Yellow mesh represents anomalous difference electron density map contoured at 4 σ. Superposed L-proline (from the *Mt*P5CR/L-proline complex, wires) is shown to visualize the Cl^−^ overlapping the carboxyl O. **(B)** Superposition of *Mt*P5CR/L-proline and *Mt*P5CR/NAD(P)^+^ complexes reveals that the MOPS molecule (originating from NAD(P)^+^ complexes) partially overlaps with L-proline (wires).

*Mt*P5CR crystallized in the presence of Mg^2+^ and Ca^2+^, but neither cation was found in the electron density maps of *Mt*P5CR structures. On the contrary, in the crystal structures of unliganded *Mt*P5CR and both holo complexes, chloride anions are present in the catalytic center (Figure 9A). Examination of the vicinity of the chlorides revealed up to seven (chain- and structure-dependent) H-bonding interactions that, in the complex with L-proline, are distributed between its two carboxyl O atoms. Up to four of these bonds are formed with N atoms. Also, highly positive peaks in *F*_*o*_–*F*_*c*_ electron density maps appear if a water molecule is placed instead of the chloride, suggesting a larger atom. The presence of chlorides was verified with the use of anomalous difference electron density map, resulting from diffraction data collected at 1.7196 Å wavelength (Figure 9A).

### Binding of anions that partially overlap with the proline binding site can result in inibition of *Mt*P5CR activity

In the catalytic center of the enzyme, a MOPS molecule was also found in the structures with either coenzyme. Superposition of *Mt*P5CR structures complexed with L-proline and NAD(P)^+^ revealed that such a molecule is located between the product and the coenzyme (Figure 9B). In each case it is stabilized by a single hydrogen bond between the O atom from a MOPS sulfate group and O2′ of the nicotinamide-adjacent ribose. Upon superimposition with the *Mt*P5CR/L-proline complex, the morpholine ring of MOPS partially overlaps with proline, although not interacting with the protein residues that establish contacts with the amino acid. As a result, the distance between the nicotinamide C4 atom of NAD(P)^+^ and Cδ of proline in the superposed structures is about 5.1 Å, whereas in the structure from *S. pyogenes* this distance is about 3.3 Å (Nocek et al., 2005).

**Figure 10:**
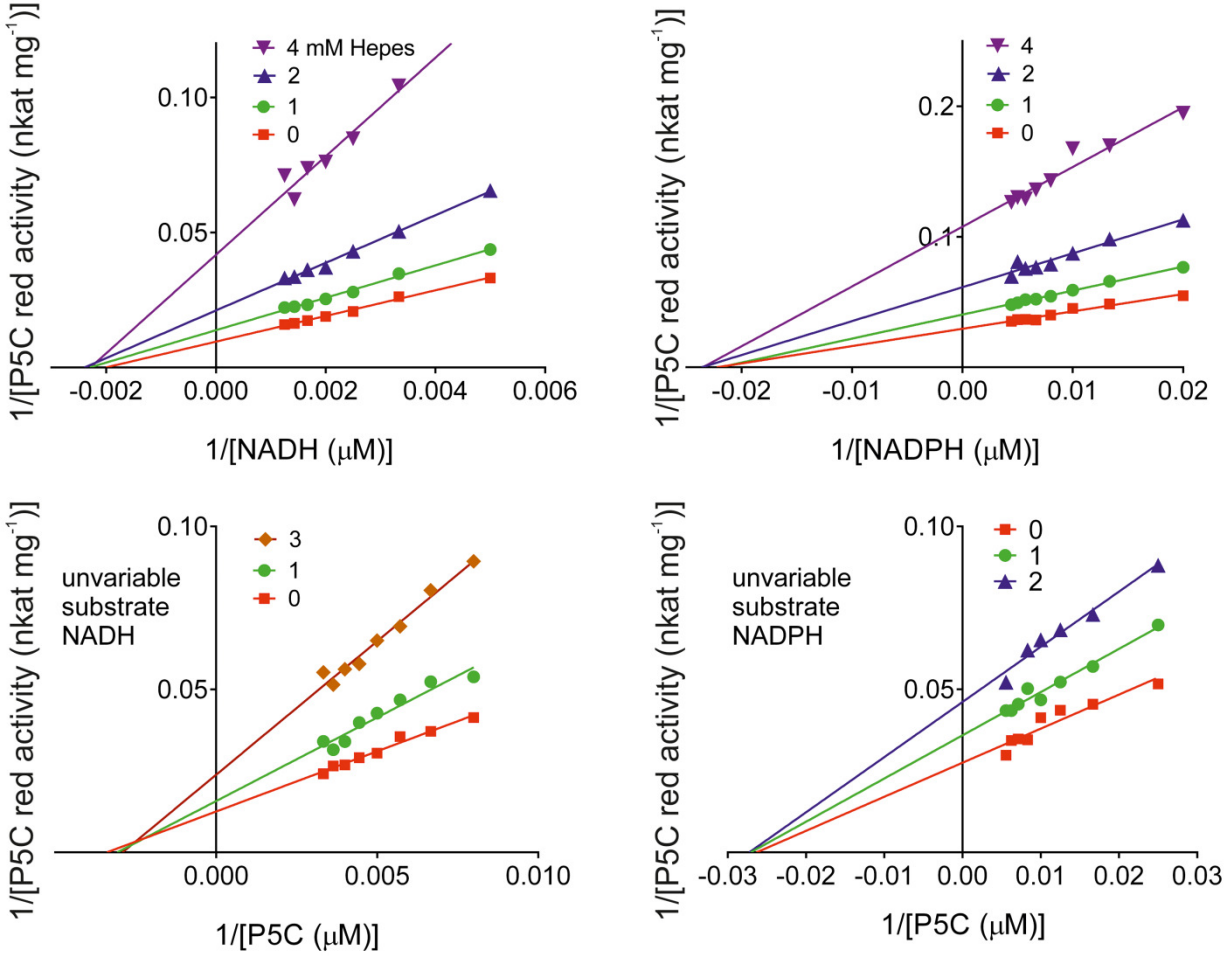
**Kinetic analysis of the inhibition of ***Mt***P5CR by HEPES**. Enzyme activity was measured at varying substrate concentrations in the presence of various inhibitor levels. Invariable substrates were fixed as in the standard mixture. Three replications were carried out for each treatment. Lines intersecting at the x-axis in the Lineweaver-Burk plots accounted for non-competitive inhibition types in all cases. Non-linear regression analysis of data [Michaelis-Menten fit, Lineweaver-Burk (double-reciprocal) transform, linear regression over Michaelis-Menten data, and non-competitive inhibition] was computed using Prism 6 for Windows, version 6.03.

Because of its positioning inside the catalytic center of *Mt*P5CR, the effect of increasing concentrations of MOPS on enzyme activity was evaluated. Quite surprisingly, in the 1–100 mM range MOPS did not cause any significant inhibition. However, another commonly used sulfonic buffering agent, HEPES, progressively inhibited both the NADH- and the NADPH-dependent activity of the enzyme, with an IC_50_ value of 3.0 ± 0.2 mM. Kinetic analyses (Figure 10) were consistent with inhibition by a non-competitive mechanism with respect to all substrates, with K_I_ values of 1.83 ± 0.08, 1.91 ± 0.06, 2.84 ± 0.17, and 3.10 ± 0.36 mM for NADH, NADPH, P5C _(NADH)_, and P5C _(NADPH)_, respectively.

## Discussion

### The availability of the three-dimensional structure of plant P5CR allows a better comprehension of the catalytic mechanism and the regulatory interactions that modulate its activity

This is the first paper to describe the structural characterization of P5CR from a plant. Four crystal structures -unliganded *Mt*P5CR, its complexes with NAD^+^ and NADP^+^ and its complex with L-proline- revealed a structural model for the *M. truncatula* enzyme. Given that the plant proteins share a high level of sequence similarity (Forlani et al., 2015b; this work, Figure 2), at least part of these results can be extrapolated to other plant species. Indeed, with the only exception of the sensitivity to product inhibition by proline, also the biochemical and kinetic properties of *Mt*P5CR were found similar to those recently pointed out for the enzyme from *A. thaliana* (Giberti et al., 2014) and *O. sativa* (Forlani et al., 2015a).

For this study, the reaction product (L-proline) was used instead of the substrate (L-P5C) to determine the active site of *Mt*P5CR, due to the high lability of P5C at neutral pH values (Williams and Frank, 1975). Upon binding, the N atom of proline forms a weak hydrogen bond with Oγ of Thr243. It is, however, possible that P5C can create a different interaction involving its N atom. This is because the imine N atom in P5C has sp^2^ hybridization, which means that the N-bound hydrogen would point toward the carbonyl of Val236 and contribute to increased affinity for P5C vs. L-proline. This interaction could explain why *Mt*P5CR and some other P5CRs are not subjected to product inhibition by L-proline (Merrill et al., 1989; Petrollino and Forlani, 2012). Higher affinity for the substrate than for the product would be very beneficial, as proline may be very abundant in the cell, mainly under stress conditions. The crystal structure of the only other P5CR/L-proline complex available to date, that of *Sp*P5CR (Nocek et al., 2005), strengthens the hypothesis that in the active site of *Mt*P5CR P5C can interact with the carbonyl of Val236 (Ile219 in *Sp*P5CR). It would be of great interest to verify whether structural differences favoring or disfavoring this interaction may discriminate proline-insensitive P5CRs [the human PYCR1 (Meng et al., 2006), *Sp*P5CR (Nocek et al., 2005), *Mt*P5CR (this work)] from those that are susceptible to proline inhibition, at least when NADH is the electron donor [the human PYCR2 (Merrill et al., 1989; Meng et al., 2006), *At*P5CR (Giberti et al., 2014), *Os*P5CR (Forlani et al., 2015a)]. Unfortunately, however, a three-dimensional structure showing a detailed proline binding mode by a proline-sensitive P5CR is still unavailable.

Because of the presence of a MOPS molecule in both complexes with coenzymes, interpretation of the reaction mechanism based on the presented structures is not straightforward. It is, however, possible to analyze the substrate and coenzyme binding order. According to Nocek et al. (2005), in the *Sp*P5CR/L-proline complex the entrance to the catalytic center is blocked when a coenzyme is bound by the N-terminal domain. Therefore, P5C would be required to be bound prior to NAD(P)H. However, from crystal structures of *Mt*P5CR/NAD(P)^+^ complexes we learn that the channel leading to the P5C-binding cavity is relatively wide (about 10 Å). This means that, at least in *Mt*P5CR, the substrate can easily penetrate its way to the catalytic center of a protein molecule whose dimer mate has already bound the coenzyme.

### Consistent kinetic and structural data suggest that NADPH may be the only electron donor *in vivo*

P5CRs from most sources were reported to be able to use *in vitro* either NADH or NADPH as the coenzyme, with contrasting affinity and maximal activity. However, recent biochemical data questioned the possibility that NADH can be used *in vivo* (Giberti et al., 2014; Forlani et al., 2015a). The present results strengthen this conclusion, as the highly conserved L3 loop was found to strongly interact with the O2′-ribose phosphate group of NADP^+^, implying a higher affinity for NADPH. Since the crystallization is a long-term experiment, the oxidized, more stable forms of the dinucleotides were used instead of the hydride donors. Both NAD^+^ and NADP^+^ coenzymes are bound by the N-terminal domain of *Mt*P5CR. The description of binding modes is based on chains A, as they have the best-quality electron density maps for the L3 loops. Nevertheless, a majority of the binding contacts are preserved in other subunits and the small discrepancies can be attributed to different solvent exposures within the crystal lattice. The L3 loop is the most flexible structural element of *Mt*P5CR. In apo structures (unliganded and complexed with L-proline) loop L3 occupies a part of the coenzyme-binding site. Moreover, in these two complexes the imidazole ring of His45 lies in a structural position similar to that of adenine from NAD(P)^+^. Binding of a coenzyme undoubtedly stabilizes the L3 loop, and this effect is most evident when NADP^+^ is bound.

Interestingly, the sequence of the L3 loop is highly conserved among plant P5CRs (Figure 2), especially a histidine residue at position 45 (in *Mt*P5CR), which is present in all compared plant orthologs. It is worthy to note that the corresponding sequence motif in rice has even more positively charged residues that could interact with the phosphate of NADPH. Consistently, the difference between the sensitivity to NADP^+^ of the NADH-dependent and the NADPH-dependent activity is higher for the rice enzyme (103-fold; Forlani et al., 2015a) than for *Mt*P5CR (26-fold; Figure 3). Even though in the spinach P5CR the histidine is the only amino acid able to carry positive charge and serve as H-bond donor within the L3 loop, the enzyme showed a 10-fold lower K_M_ for NADPH than for NADH (Murahama et al., 2001). As a consequence, NADP^+^ strongly inhibits the P5C-to-proline conversion if NADH was used as the coenzyme, whereas it is almost ineffective at physiological concentrations when NADPH is the electron donor. In alfalfa (*Medicago sativa*), NADH and NADP^+^ concentrations of 9 and 37 nmol g^−1^ (fresh weight), respectively, have been found, whereas the NADPH/NADP^+^ ratio was approximately equal to unity (Igamberdiev et al., 2004). Similar ratios have been reported in other plant species (Hayashi et al., 2005; Takahashi et al., 2009). Under these conditions, and also taking into account the high K_M_ values for NADH and P5C _(NADH)_ (Table 2), the NADH-dependent activity would therefore be negligible. This may solve substrate ambiguity, as it suggests that *in vivo Mt*P5CR most likely relies entirely on NADPH as the reducing agent.

A preferential use of NADPH over NADH may have some important implications *in planta*, assuming P5CRs are also present in chloroplasts (Rayapati et al., 1989; Murahama et al., 2001). As a result of P5C-to-proline conversion, NADP^+^ is produced. In chloroplasts, NADP^+^ can enter the OPPP, which not only regenerates ribulose-5-phosphate, but also provides fresh NADPH and CO_2_ (Kohl et al., 1988). Production of carbon dioxide is particularly beneficial during water stress conditions, when the stomata are closed, which adversely impacts CO_2_ assimilation. Therefore, owing to P5CR activity, photosynthesis can proceed and the plant does not suffer from either photoinhibition (Hideg et al., 1998) or excess reactive oxygen species in the chloroplast. However, the subcellular location of P5CRs *in planta* is still under debate, as no universal pattern has been determined. Studies on soybean (*Glycine max*) revealed P5CR activities in both cytosol and plastid fractions (Kohl et al., 1988; Szoke et al., 1992), while the enzyme from pea (*Pisum sativum*) (Rayapati et al., 1989) and the spinach P5CR isozyme 2 (Murahama et al., 2001) were located in chloroplasts.

### Anions but not cations seem likely to modulate *Mt*P5CR activity

The effects of Cl^−^ ions on *Mt*P5CR activity were evaluated by *in vitro* tests, which showed that high concentrations of chlorides are inhibitory. Conversely, low chloride concentrations had a stimulatory effect on the NADPH-dependent activity. These results are consistent with those recently obtained using P5CRs from *A. thaliana* (Giberti et al., 2014) and rice (Forlani et al., 2015a), although in those cases enzyme stimulation by chlorides was found only with NADPH as the electron donor. Such a feature is extremely interesting from a physiological point of view, since it provides a rapid way to increase hyperosmotic stress-induced proline synthesis in the presence of excess chlorides in the cytosol, without the need for transcriptional control of *P5CR* expression. However, the mechanism by which Cl^−^ exerts its action has never been explained before. In the crystal structures of unliganded *Mt*P5CR and both holo-complexes, chloride anions were found in the catalytic centers at the same structural positions as the carboxyl groups of L-prolines in the *Mt*P5CR/L-proline complex (Figure 9A). Therefore, as inferred from the crystal structures, chloride ions should be rather inhibitory as they compete for the same site with the carboxylic group of L-proline and, most probably, P5C. Indeed, the concentration of Cl^−^ ions in the crystallization drop when the crystals were harvested should have exceeded 340 mM. As a consequence, we propose that the Cl^−^ found in the crystal structure reflects the inhibitory role of Cl^−^.

It is, on the contrary, difficult to determine the mechanism of stimulation of enzyme activity by a low concentration of chloride ions because there are no more Cl^−^ binding sites found in the crystal structures of *Mt*P5CR. The *stimulatory* Cl^−^ could bind to the same site and, in that way, keep the L-proline (and P5C) binding site, the L19 loop, properly arranged (Figure 9A). Perhaps, in the absence of Cl^−^, the loop incorporates a water molecule, changes conformation and becomes unable to bind P5C. In that scenario, it would be only a matter of shifting equilibrium, where at low (stimulatory) concentrations of Cl^−^ the P5C can enter the reaction venue, whereas at high (inhibitory) concentrations the halogen prevents P5C from entering. This is, however, merely speculation, and additional tests, e.g., with other Na-halogen salts that would differently modify the L19 loop, will be required to shed further light on the mechanism. *In planta*, where local chloride concentrations in the range of 25–100 mM can be considered physiological in salt-stressed plants (Britto et al., 2004), only the *stimulatory* role of chloride ions seems relevant. On the contrary, chloride concentrations at which *Mt*P5CR activity is inhibited are far from occurring even in plants exposed to extreme salinity.

On the other hand, and despite their presence in the crystallization medium, neither Mg^2+^ nor Ca^2+^ ions were found in the electron density maps of *Mt*P5CR structures. Interestingly, both cations were reported as inhibitors of P5CR from spinach, against which divalent cations were found stronger inhibitors than monovalent cations (Murahama et al., 2001). However, in that study all cations were added as chloride salts. It is, therefore, impossible to distinguish between what actually worked as an inhibitor, the divalent cations or the chloride anions, especially when divalent salts dissociate to double the Cl^−^ concentration. Additionally, the divalent cations are intrinsically prone to complexing with the pyrophosphate group of NAD(P)H, and metal-complexed NAD(P)H cannot enter the binding site. Consistently, in the case of spinach P5CR, ATP was found inhibitory by competing with NAD(P)H, and the inhibition was partially mitigated when MgCl_2_ was also added (Murahama et al., 2001). Most likely, in that case some ATP complexes with Mg^2+^ cations and, therefore, ATP cannot bind to the NAD(P)H binding site and cannot inhibit P5CR.

### The presence of anions in the catalytic center and their effects on enzyme activity provide a new lead for the development of effective inhibitors of P5CR activity

In the complexes with either coenzyme, a MOPS molecule was found in the catalytic center of the enzyme. As a result, the distance between the nicotinamide C4 atom of NAD(P)^+^ and Cδ of proline in the superposed structures increases from 3.3 Å in *Sp*P5CR (Nocek et al., 2005) to about 5.1 Å. A short distance should allow *in vivo* a direct transfer of the hydride from the *pro*-S face of C4 of NAD(P)H to C5 of P5C. To verify this hypothesis for *Mt*P5CR, a different crystallization condition, without MOPS buffer, needs to be developed. It is possible, however, that binding of MOPS actually enables the crystal formation, as it forces the protein molecules to adopt the same conformation. Only in the structure with L-proline, which was obtained *via* soaking, MOPS is not present, but its coenzyme-binding domain is shifted by less than 0.4 Å when compared to the NAD(P)^+^-bound complexes. Apparently, prior to soaking the protein molecules had adopted the crystallization-promoting conformations, which were maintained by inter-decamer contacts in the crystal lattice and were not changed by subsequent product binding.

Whatever the mechanism, the binding of anions near the L-proline binding site may have interesting practical applications. The addition to the assay mixture of MOPS concentrations up to 100 mM was found ineffective. However, when HEPES was used instead, which bears a larger 2-[4-(2-hydroxyethyl)piperazin-1-yl]ethane moiety than the 3-morpholinopropane moiety of MOPS, a significant inhibition of enzyme activity was found. On the whole, the inhibitory effect is mild, and evident only at relatively high concentrations. However, the mechanism of the inhibition by HEPES is very interesting because, as a non-competitive inhibitor, it does not depend on the concentration of enzyme substrates.

Being placed at the converging point of the two pathways leading to proline synthesis in plants and most microorganisms, P5CRs are attractive targets for the development of either new therapeutics (Forlani et al., 2012) or new active ingredients for weed control (Forlani et al., 2007, 2008). Several recently identified aminomethylene-bisphosphonic acids are able to inhibit plant P5CRs at micromolar concentrations. However, the most active compounds showed an uncompetitive type of mechanism with respect to P5C (Forlani et al., 2007). As a consequence, they would be most effective in the presence of saturating levels of P5C, which is, on the contrary, maintained at very low concentrations inside the cell (Forlani et al., 2013) to avoid cytotoxic effects (Deuschle et al., 2004). In the presence of limiting P5C concentrations, interaction with such inhibitors results in a higher affinity for the substrate. This would cause a lower effectiveness *in vivo* than *in vitro*. Moreover, because the crystal structure of the plant enzyme was unavailable at the time, aminophosphonate inhibitors were designed using a computer-aided docking procedure performed on the basis of the crystal structure of the enzyme from *S. pyogenes* (Nocek et al., 2005). Consistently, the susceptibility of the bacterial enzyme has been found to be strikingly higher than that of the plant P5CR, with IC_50_ values differing by 2-3 orders of magnitude (Forlani et al., 2012, 2013). The present results, therefore, provide new information on these aspects, and point at the alkylsulfonic acid moiety as a new lead for the development of specific inhibitors targeting plant P5CR.

## Conclusions

This work was focused on description of the main features of a plant P5CR from a functional and structural perspective. The enzyme from the model legume, *Medicago truncatula*, was chosen for the study. Four crystal structures (unliganded *Mt*P5CR, its complexes with NAD^+^, NADP^+^, and L-proline) were solved and provided a structural model for the protein. Even though P5CR sequences differ between species from different domains of life, we showed that the overall fold of *Mt*P5CR is similar to those of the bacterial and human orthologs, which have had their three-dimensional structures determined (Nocek et al., 2005; Pike et al., unpublished).

The coenzyme preference for NADPH over NADH, reported previously for other plant species (Szoke et al., 1992; Murahama et al., 2001; Giberti et al., 2014) based on enzymatic assays, was confirmed and interpreted for the first time from the structural point of view. The highly conserved L3 loop was found to strongly interact with the phosphate group connected to O2′-ribose of NADP^+^, implying a higher affinity for NADPH. This observation was also supported by enzymatic assays, which showed a 12-fold lower K_M(app)_ value for NADPH than for NADH. Moreover, NADP^+^ was found to strongly inhibit the P5C-to-proline conversion if NADH was used as the coenzyme, whereas NADP^+^ was almost ineffective at physiological concentrations when NADPH was the hydride donor. NAD^+^ did not exert any inhibitory effect regardless of the coenzyme used. Therefore, considering the physiological concentrations of reduced and oxidized forms of both coenzymes, it is highly unlikely that NADH could serve as the reductant *in vivo*.

Last, but not least, the effects of chloride ions, MOPS, and HEPES molecules on the enzyme activity were tested. Chloride ion was found to occupy the same position as the carboxyl group of L-proline. In *in vitro* tests low concentrations of chloride ions had a remarkable stimulatory effect on the NADPH-dependent activity, whereas high Cl^−^ concentrations inhibited *Mt*P5CR. Interestingly, HEPES in the millimolar range strongly inhibited the reaction. Therefore, we propose HEPES as a scaffold for designing new P5CR inhibitors. Given that plant P5CRs share high sequence similarity, many results, obtained within the scope of this project, can be extrapolated to other plant species.

## Accession numbers

Coordinates and structure factors of the related structures were deposited in the Protein Data Bank (PDB): *Mt*P5CR-unliganded, PDB ID: 5bse; *Mt*P5CR-NAD^+^ complex, 5bsf; *Mt*P5CR-NADP^+^ complex, 5bsg; *Mt*P5CR-Pro complex, 5bsh.

### Conflict of interest statement

The authors declare that the research was conducted in the absence of any commercial or financial relationships that could be construed as a potential conflict of interest.
